# Knowledge of and preferences for health insurance among formal sector employees in Addis Ababa: a qualitative study

**DOI:** 10.1186/s12913-015-0988-8

**Published:** 2015-08-11

**Authors:** Amarech Obse, Damen Hailemariam, Charles Normand

**Affiliations:** School of Public Health, College of Health Sciences, Addis Ababa University, Addis Ababa, Ethopia; Centre for Global Health, Trinity College Dublin, 3-4 Foster Place, Dublin 2, Ireland

## Abstract

**Background:**

The Ethiopian health system has been undergoing through reforms. One of the reforms stipulated in policy documents is the introduction of health insurance at national level. Having the majority of the population without any experience of health insurance, investigating preferences and knowledge of the essence of health insurance among potential enrolees will provide vital information for policy makers. This formative study seeks to explore the knowledge and the preference for health insurance among formal sector employees in Addis Ababa.

**Methods:**

Six focus group discussions with formal sector employees and five key informant interviews were conducted in Addis Ababa. A thematic analysis is used to analyse the results.

**Results:**

The findings suggest that there is little knowledge about the concept and elements of health insurance. Some concepts such as, risk pooling and sharing are not well understood. The participants of the study considered health insurance as only a prepayment mechanism without risk sharing among members of the scheme. Regarding preference for health insurance, they have revealed quality of care as the most important factor. Comprehensiveness of benefit packages and the amount of premium level are also found to be concerns related to health insurance. However, a trade-off is also observed among premium level, comprehensive benefit packages, and healthcare facilities.

**Conclusions:**

Improvements on availability and quality of services need to precede the introduction of social health insurance. There is also a need to work on awareness creation regarding concepts of health insurance. Further studies may explore if the knowledge gap is real or appeared due to reservations of the participants on the introduction of health insurance.

## Background

In the context of low income countries, low understanding and knowledge of the notion of health insurance had been contributing to low level of enrolment to voluntary health insurance schemes. During sensitization and awareness creation for health insurance interventions, the focus is usually on the amount of premium that potential enrolees are expected to pay. Less focus on explaining concepts such as solidarity, risk pooling, moral hazard, and adverse selection is a limitation [1, 2].

Apart from misunderstanding health insurance concepts, scepticism and distrust of new health insurance schemes is also a reason for declining the initiatives. Sentiments as a result of bad experience with other solidarity arrangements affect the attitude towards health insurance initiatives. In addition, lack of confidence in institutional capacity, the ability of management to follow through, perception, attitude [2–4] and cultural resistance related to earmarking resources for health care influence the demand for health insurance [5]. Though individual attitude and valuation of health insurance is fundamental to participation in voluntary health insurance, it also has important implications to compulsory health insurance schemes [1].

Irrespective of their health status, people prefer high quality services. Perceived quality of health care services is explained by different aspects of providers [6]. As users of services, consumers are the ones who assumed to know the qualities. They also seek low cost services since they do not want to make expensive payments. As a result, consumers’ health plan choice is recognized as a key indicator of high quality and low cost services [7]. Dissatisfaction with quality of care and competence of health care personnel are among the factors which influence the preference for health insurance [8].

Other factors which determine the preference for health insurance include benefit package, enrolment, copayments, and other features of the scheme [2, 9]. The pattern of preference is also influenced by socio demographic factors such as gender, wealth status/income, health risk, and age [10–14].

The World Health Report 2000 states that health systems should be responsive to people’s expectations [15]. However, the poor population segments in low income countries can only buy severely rationed health insurance packages. Consequently, they are exposed to high potential distress due to the limited access to health care since they are not involved in the allocation decisions of health care interventions [16, 17].

This study used qualitative investigation of the knowledge and preferences of formal sector employees for social health insurance (SHI). The study aims to generate an understanding of potential enrolees’ knowledge and preferences which will be useful for policy-makers in the country.

## Methods

### Setting

The Ethiopian health system is financed from multiple sources. Out of pocket payments, general taxation, and donation are the main mechanisms of financing. The system is highly dependent on out of pocket payment and thus suffers from under-financing. Health insurance and prepayment mechanisms have been almost non-existent [18]. This has resulted in equity problems since there are no effective fee-waiver and exemption systems [19].

The National Health Accounts (NHA) [20–24] show a general increase in health care expenditure both in nominal and real terms. Per capita health expenditure grew from US$7.1 in 2004/05 to US$20.8 in 2010/11. Most of the increase in health expenditure came from households and donors. Donors, households, and government respectively contributed 49.9, 33.7, 15.6 % of health spending in 2010/11.

In order to alleviate the health care financing challenges, the Federal Ministry of Health developed a health care financing strategy in 1998 [25]. One of the strategies stipulated was the initiation of various types of health insurance schemes. Following this the health insurance strategy was developed in 2008 to introduce health insurance at national level. As specified in the policy documents, all formal sector employees will be covered by compulsory social health insurance. The scheme will be first introduced among civil servants and an incremental approach will be used to expand the coverage for all formal sector employees. Moreover, informal sector employees will be covered by community based health insurance which will be scaled up following a pilot study. To that effect, many activities and studies were conducted to supply information on different dimensions of health insurance.

This study is part of a wider study which assessed the demand for health insurance among formal sector employees. This is a formative study which sought to explore the knowledge and preferences of potential enrolees of SHI. Low understanding of health insurance schemes due to lack of experience or other attitude or culture related factors prevail in countries such as Uganda and West Africa [2–4]. The Ethiopian health system is undergoing reforms on which is the introduction of social health insurance at national level [26]. Having no such scheme before, there is a need to investigate the knowledge of and preferences for health insurance schemes among potential enrolees. This study is led by the question “what knowledge does the formal sector employees have regarding health insurance and what design characteristics do they prefer?”

### Participant selection

A purposive sampling strategy is used to selected participants with and without health insurance. Similarly, respondents for the KIIs are purposively selected considering their experience working in health care financing reforms or insurance. Three FGDs were conducted with civil servants who did not have experience of any type of health insurance and the remaining three were conducted with employees’ of public enterprises who benefit from employment based health insurance (EBHI).

In order to select participants for FGDs, first the organizations were identified. Ten (five public enterprises and five civil service) organizations were selected. Since the target populations for this study are formal sector employees, they were contacted at their workplace during working days. Considering that the FGDs were to be made at working hours, the mangers of 3 organizations declined the request. They suggested the FGDs to be conducted off working hours. However, it was not possible to bring together workers for the FGDs off working hours since they will be leaving. Therefore, the organizations which refused the FGDs were replaced by others which allowed the FGDs during working hours. The proposed number of discussants for the focus groups was 6–8 [27]. They were selected with the help of a focal person assigned by the managers of the organizations. Depending on the size of the organization the number of participants from each department differed. In big organizations departments were first selected. Then, willing participants (1–2) from each department were selected. The focal person made announcement in selected departments and asked for individuals who were willing to participate in the FGDs. In one organization, the mangers did not allow two workers in specific department to participate in the FGDs indicating that they were expected to complete a given task. In another case, none of the employees willing to participants from one of the selected department and needed to be replaced by another department. The main reason for refusal by employees was availability of tasks due. The key informants were selected from the insurance corporation and the Ministry of Health who worked on health care financing reform.

### Data collection and analysis

The knowledge and preferences regarding SHI among formal sector employees was explored through 6 focus group discussions (FGDs) and 5 key informant interviews (KII). The FGDs are conducted in the rooms provided by the managers at the work place of the participants. The interviews were also conducted at the offices of the key informants. Before the FGDs and interviews started, the researchers introduced themselves and explained the purpose of the study. They asked the willingness of the participants and key informants and ensured them that they can stop the interview or leave the discussions at any time. FGDs and KIIs were conducted after verbal informed consent was obtained from all participants.

The interviews and discussions were started with the introduction of the participants. The participants introduced their names, work experience and positions, and health insurance status. The authors met all participants of the FGDs for the first time. Both the FGDs and KII were conducted using guiding questions specifically prepared for the discussions and interviews. The guiding questions structured the information that was collected though thematic areas were explored freely as they arose. Guiding questions were prepared by the first author and revised by co-authors. The interviews and discussions focused on the following issues: concept of health insurance, solidarity, risk-pooling, and co-payments; benefit packages; health care providers’ choice; and premium levels.

The FGDs and KIIs were conducted by the corresponding author with an assistant. All FGDs and four of the five KIIs were tape recorded. The FGDs and the key informant interviews were conducted between January and February 2012. The FGDs lasted between 1:30 – 2:00 h while the key informant interviews took 0:30 – 1:00 h. The interviews and the FGDs were conducted in local language. They were transcribed verbatim and translated to English. Manual thematic analysis [28] was used to analyse the findings.

Ethical clearance for this study was given by the Institutional Review Board (IRB), College of Health Sciences, Addis Ababa University and a research ethics committee of Centre for Global Health, Trinity College Dublin.

## Results

The focus group discussions included a total of 43 participants, out of which 27 (63 %) were male. Three of the FGDs consisted of 8 participants, two of which comprised 6 participants and one had 7 participants. The participants of the FGDs were mainly mid-level to low level workers. The work experience of the participants ranged from 1.5–15 years. All of the key informants were male who were involved in health insurance and health financing reforms. Their work experience raged between 7 and 15 years.

### Knowledge of health insurance

The knowledge regarding health insurance was explored in three areas: knowledge of the essence of health insurance, knowledge about the available health insurance, and knowledge on health insurance proclamations.

The key informants revealed that there is medical insurance which is provided by a handful of commercial insurance companies. However, most participants of the FGDs were not aware of the availability of such scheme apart from people who are insured by their employer. None of those uninsured purchased insurance policies for themselves. The clients of medical insurance in the country are mainly organizations who purchase medical insurance on behalf of their employees. Some civil servants are aware that there is a privilege of employment based health insurance for those employed in public enterprises.

The overall discussion about health insurance showed that there is some understanding of what health insurance is. However, there is very limited knowledge about some concepts of health insurance such as risk pooling and cost sharing even among those who are currently insured. Most of the participants from civil service organizations were found to be against cost sharing. They believed that it is the responsibility of the government to provide health insurance. The justifications used were the type of EBHI whereby private employers cover the total health expenditure without contributions by the beneficiaries and with no co-payments in most cases. Similarly, participants from the public enterprises were not happy about the cost sharing and the cap.

*“Government should provide us full health insurance because we do not have the ability to pay. I know private employers provide health insurance for their employees. Why not the government provides full insurance for its employees?”(female respondent, uninsured)*

Among the concepts of health insurance, risk pooling is not well understood. Some of the participants revealed that they would like to be paid back the sum of the contributions they have made at the end of a year if they did not fall sick within that year.

*“Health insurance is good, I will be sure that I have money when I get sick. So I am safe. But what if I didn’t get sick within a year? I do not want to keep paying for health insurance scheme if I do not get sick and I need to be paid back the sum of money I put away at the end of the year if I did not fall sick” (male respondent, insured)*

Most of the participants heard about the new social health insurance proclamation but only few had further information. The majority of the participants were happy about it and appreciated the effort. Others question whether the objective of health insurance is to address financial inaccessibility to health care or to be another source of revenue for the government.

Key informants were asked to share their knowledge on the low development of health insurance in Ethiopia and why some employees used to be insured while others were not. They revealed that health benefits were not available for civil servants in part because they were not aware of these benefits. However, still there were challenges of infrastructure to provide services even if there was wish to provide them. Otherwise, proclamation 515/2007 entitles civil servants to get access to health care with a prepayment mechanism.

*“It (proclamation number 515/2007 on health benefits) was not operational because the civil servants do not know that they have this right and they didn’t ask for. Even if the civil servants demanded the service, the facilities were not in a position to provide that service. There was only proclamation but it was not clear how the services should be provided. For instance which services should be provided? Are drugs included? Or is it only for consultation? So, nobody saw this being practiced; may be some civil servants had benefited. There was also no work from the government side to let the civil servants know that they have this benefit…” (key informant from MOH)*

As stated by the key informants, the reason for the new reform was under-financing of the health system as shown by the NHA of 1995/96 [20]. Thus, there was need for resource mobilization and sharing of healthcare costs. In addition, there was need for new proclamation since the previous one did not work.

With regard to medical insurance provided by commercial insurance companies, the clients are mainly organizations that purchase the scheme on behalf of their employees. Only very few people purchase this medical insurance personally. These people are usually found to be well educated and in a relatively high social class. On the other hand, the insurance companies are reluctant to promote and expand the provision of medical insurance since it is not profitable. The premiums are high and are not affordable to the general public. Moreover, there are many restrictions in terms of benefit packages, payment cap, and co-payments. Claim management of medical insurance was said to be the most important factor for the companies that make the provision of medical insurance expensive.

*“…we are profit making company and in case of medical insurance there are many claims compared to other types of insurance… there are lots of claims for medical insurance per year…” (key informant from insurance corporation)*

The key informants indicated that there is still a view that Ethiopia is not yet ready for health insurance considering the capacity of the health system coupled with ever increasing population pressure. However, there will still be a need to start health insurance somewhere and use incremental approach to expand coverage by learning from practices.

### Preference for health insurance

Once the knowledge of the participants regarding health insurance was explored and awareness was created, discussions were made regarding the factors that are important in their preference of health insurance. The main health insurance design elements raised to be most important are monthly contribution rate, benefit packages, quality of services, health care providers, enrolment, co-payment, and payment mechanism.

### Premium

In spite of the appreciation of the initiation of social health insurance (SHI), the majority of participants for the FGDs both from public enterprises and civil service organizations were not happy about making contributions for the scheme. But further discussion regarding health care resources provided awareness and consensus that there should be contribution by beneficiaries. Nevertheless, the civil servants remained concerned and reluctant. They stressed that it would be very difficult for them to make contribution for different but interrelated reasons including low salary scale, very high and ever increasing cost of living, and burden of other contributions made from their salary. Thus, they proposed a pay rise if they have make contributions.

*“… We currently make many expenses in the form of contribution… and now we are required to contribute for health insurance … in the short run we may fail to meet our basic needs… we may even fail to adequately feed our family. This indirectly leads to health problems, and lack of capacity to resist illness… The idea of health insurance is good but they need to consider civil servants’ ability to pay among different contributions and expenses with our low salary…” (male respondent, uninsured)*

Considering their views about premium, discussions were made on payment modalities of out of pocket and prepayment to further explore their demand for health insurance. Most of the participants revealed that they prefer health insurance on certain conditions such as freedom in provider choice, availability of high quality service, provision of comprehensive benefit packages, and removal of other access barriers. They stressed that in present condition paying the premium will just be additional cost and that addressing only financial barrier will not solve the problem of inaccessibility.

*“…even if the premium is deducted from your salary, you still go to other providers and pay out of pocket. So I prefer to pay out of pocket… to pay myself when I need care… I can go to the provider of my choice… so unless the services are first made accessible, it is better to leave the insurance and we pay out of pocket.” (female respondent, uninsured)*

Key informants also share this idea, and they have indicated that quality and availability of healthcare services is the very important issue that needs to be addressed. They indicated that there are different reforms in addition to the health care financing reform that is undergoing in the health sector to deal with the other accessibility problems. They also indicated that the implementation of SHI is delayed since preparatory activities had been underway to address other health system problems.

The key informants revealed that the proposed premium for social health insurance is 3 % of gross salary to be contributed by both employees and employers. Considering the current cost of service provision, the key informants considered the proposed contributions to be small and they indicated that there may not be sufficient resources mobilized to provide all the services the target population need.

On the contrary, the participants from civil service organization claim that the proposed contribution rate to be rather high:

*“The proposed amount of premium for the social health insurance is 3 %. When I see this with our utilization of health care, it is a lot. We don’t have the culture of going to health care providers unless we have serious problem. So we will only be incurring additional cost from paying the premium. So I suppose it should be between 1–1.5 %. Then, adjustments can be made through time as the utilization level increase, quality of services increase, and cost of service provision increase, 3 % is the maximum in the current situation with poor quality of health care providers, and inadequate resource.” (male respondent, uninsured)*

However, participants tended to offer higher premium levels for comprehensive benefit packages:

*“… the contribution rate should depend on the types of services covered. If there is comprehensive coverage especially for types of services which are very expensive and life threatening, 3 % is fine. Otherwise, if the coverage is only limited for some services, it shouldn’t be more than 1 %.” (female respondent, uninsured)*

Participants from public enterprises offered higher contribution rates. They offered a minimum of 3 % of their monthly salary for type of benefit package with exclusion of some chronic diseases and dental care while the civil servants offered 0.5 % for a comparable type of benefit packages.

The participants also suggested a progressive type of contribution to address the issues of social welfare in making contribution like wealth redistribution.

### Benefit packages

There is very limited knowledge about benefit packages even among those with employment based insurance. As would be expected, participants opted for comprehensive benefit packages and they did not understand why there is a need for exclusion of health care services from the benefit packages. It appeared for them as if it meant that the health of some people is more important than that of the others. Some claim that they are not in a position to offer or discuss about benefit packages and it should be determined by healthcare professionals. They argued that unless health insurance provides comprehensive coverage, they will not be protected because they do not know what health problem they will face.

*“…an illness is illness as it affects and prohibits the normal way of life why do we prioritize?” (female respondent, uninsured)*

After long discussions about availability of health care resources and the capacity of the health system, some of the participants indicated that there would be a need to identify and prioritize health care services. Most of the participants opted for full coverage of inpatient care and drug. Drugs are listed as the most important and expensive element of health care expenditure.

*“… especially drugs are expensive even if you afford to pay for the services, you may fail to buy drugs” (female respondent, uninsured)*

Some of the participants presented their arguments about benefit packages in two categories; at country level and individual level. Considering the disease burden of the country, communicable diseases were stated to be given emphasis. Communicable diseases were said to lead to economic, political and social crisis in a short period of time. Nevertheless, others mentioned the changes in disease pattern indicating that a large number of people were being exposed to non-communicable diseases. Consequently, these problems were said to be associated with more expenses and need consideration. Likewise, outpatient services with high occurrence and low cost were also said to lead to financial ruin of the users for services and be covered with co-payments. Emergencies were raised to be covered without any condition. In some cases, individual preferences were different from propositions at national level especially among people with specific personal health condition. Obviously, they opted for coverage of their conditions.

*“…it is the question of priority… in most poor countries like Africa, the main health problems are communicable such as TB, water-borne diseases, diseases related to poverty, and diseases related to personal hygiene. So there should be effective coverage for these types of problems…” (male respondent, insured)*

*“However, chronic diseases also need to be considered since they lead to substantial expenditure…” (female respondent, uninsured)*

In the identification of benefit packages the capacity of the country in service provision is also raised beside preferences. The participants discussed that unless the country have the services to provide with adequate number and specialty of health care personnel, equipment and other inputs, including the services in the benefit packages do not help. They emphasized that these issues need to be considered while defining the benefit packages.

*“…the other thing that should be considered is the ability of the country. How much can the country provide? … Do we have specialized health care personnel to provide the services which are proposed in the benefit packages? This also needs to be answered… otherwise we just give the right to the people and there are not services to utilize.” (male respondent, uninsured)*

The benefit packages offered by employment based health insurance of public enterprises were raised for discussion. Both the beneficiaries and civil servants question the basis for the exclusion of vision and dental care which is the case in most schemes. They argue that the exclusions did not consider the disease burden of the country.

*“…in case of our country, eye illness is common. After some age, most people get eye diseases maybe because of the dust or lack of proper hygiene. There is great chance of acquiring eye disease. I don’t know why vision care is excluded from our health care benefit. I think it would have been better if they exclude many other services and include vision care.” (male respondent, uninsured)*

The proposed exclusions from social health insurance which includes cosmetic surgery, implantation, transplantation, dialysis, dental care, eye glasses and other medical aids, transportation cost were also forwarded for discussion. The participants of the FGDs agreed that there should be some exclusion because resources are not adequate.

“*the luxurious services such as cosmetic surgery can be excluded but dialysis and dental care should not be…”(female respondent, uninsured)*

Rather than identifying the benefit packages some participants preferred to state the cap that can be paid per person per year. They were reluctant to discuss priorities because they do know what kind of health problem they will face.

*“Specifying the benefit packages by services may not be important because one person may not be affected by the same health problem every time. So I think it is better if we can just decide the maximum coverage in terms of money and then people can utilize any type of service they need until the money is fully utilized…”(male respondent, uninsured)*

There was also a perception that social health insurance is there to address work related injuries only and that some of the participants stress full coverage for work related health problems. They were not sure if they would be covered for other health problems.

Moreover, participants proposed the opening of private clinics by the organizations which have capacity as an alternative. In doing so, organizations will have better capacity to provide services and there may not be need to have exclusions. Employees will only be referred for problems beyond the capacity of these private clinics. But some other participants revealed their frustration if these private clinics are the only primary service providers to the employees and may not be accessible during emergencies.

### Healthcare providers

Participants revealed that health care seeking is complicated due to service providers. They expressed their dilemma about providers.

*“Seeking treatment is difficult… if you go to public providers you will not get the services and if you go to private providers you get the services but it is expensive…” (female respondent, uninsured)*

The most important factor which made participants to hesitate about health insurance was the perceived low quality of services especially in public providers. They questioned if it is manageable to provide good quality healthcare service with the current status of health system.

*“… in this current situation of health facilities, I don’t need health insurance”(male respondent, uninsured)*

*“… With the current situation, it cannot work. We need to improve the system first: the health care providers, type and quality of the services provided and many other concerns need to be addressed. The government may solve the financial barrier but what about the waiting time? How can it ensure that I am getting the right service I need? Still, I am not concerned about the payments I make especially in public facilities but the quality of the service…”(male respondent, uninsured)*

Most participants rated the services of private facilities to be of good quality and that they prefer to get services from those facilities. They questioned whether they can get services from private providers once they are enrolled to the social health insurance.

*“…with current situation, it is difficult to get services from government hospitals…in terms of waiting time, accessibility, quality… so private facilities provide quality services”(female respondent, uninsured)*

They believed that the private providers are more accessible and they should be given the mandate to provide the social health insurance. However, there was also a view that such mandate should be provided to government facilities since they would be accountable and that the private facilities may abuse the scheme. They also pointed out that the scheme may go bankrupt if it is left to private providers since the services are expensive. All agreed that, primarily capacity should be built for the public providers in terms of quality improvement. Then, services could be provided by mix of public and private providers. Some participants also appreciated the improvement in service provision of some public hospitals in Addis as a result of large scale health sector reform. However, the services of health centres were said to be still questionable. They revealed that there were no adequate professionals at health centres which compromise the services that will be provided to them.

*“…private providers may be involved to some extent but predominantly the services should be provided by government facilities” (male respondent, uninsured)*

*“The services should be provided both by private and public providers. When we prefer private, we consider quality and waiting time… but now we also need to consider their expense. We do not need as such luxurious hospitals but those who provide quality service with modest payment.” (female respondent, uninsured)*

Respondents both from the public enterprises and civil servants underline that the premium should depend on the benefit packages and type of provider. But they agree that it is not the type of provider but the type of service that should determine the premium level.

### Enrolment

The type of enrolment at organizations which currently provide health benefits was criticized because most of them give coverage for the staff only and not for their families. There were also people who experienced the downgrading of the enrolment.

*“I was supposed to get my families enrolled then they changed the policy and they (my families) are no longer in the health benefit. So I have to pay myself if my wife or children are sick”(male respondent, insured)*

They considered individual level enrolment as a kind which ignored the type of strong social attachment of the population and thus unsatisfactory.

*“If they insure only me, doesn’t help me much, I may not get sick for long. But because I have many dependents some of them may get sick and I have to pay” (male respondent, uninsured)*

One of the issues that were raised regarding employment based health insurance was the practice of insuring professionals only in some organizations. Normally those who are not enrolled are the most needy ones; with low status and salary and thus employment based health insurance increases inequity. They consider this to be unfair.

*“We are working in the same organizations but we are insured and they are not, this is not fair”(male respondent, insured)*

Moreover, delays in the enrolment of some employees who were entitled to health insurance was also raised as one of the factors leading in to dissatisfaction. As a result, they are denied reimbursements and were paying for healthcare out of pocket.

*“I do not want to go into the details… we are entitled to the benefits but every time we ask for enrolment they give us different reasons not to enrol us”(male respondent, uninsured)*

### Co-payments and payment mechanism

The participants of the FGDs with employment based health insurance revealed that they have some kind of financial protection from health care expenses. However, they are not comfortable with the procedures of getting the insurance. Most organizations that provide employment based health insurance mainly use retrospective reimbursements to individuals; that is, employees are expected to pay the health care expenditure when they need care; then, they request for reimbursements from their organizations or the insurance companies.

*“…the objective of health insurance is to secure employees when they get sick. But in our case, first you have to pay it out of pocket, but you may not have money in your pocket. It was rather good if we could get the right to medical care from the providers. For me, it doesn’t make much difference to recover the money latter or not. I think the essence of insurance is lost if you are paying out of pocket at the time you need care.”(male respondent, insured)*

All respondents share this idea and they pointed out that they are not happy with such arrangement. They mentioned they need the insurance when they are strained with the need for medical care and payment for the care.

*“When I am sick I have to pay out of pocket and then I will ask for reimbursements. If I do not have money at the time I am sick, I still be in trouble though I am insured. I have to find someone to borrow from. Though I get the money latter, it does not make much difference because there is also a cap and they do not reimburse more than that cap” (female respondent, insured)*

Regarding co-payments, most of the participants agree that there may be need to have co-payments for outpatient services in order to reduce moral hazard. But they point out that there should not be co-payments for inpatient care, emergencies and drugs.

*“There should be 100 % coverage for emergency. Inpatient care is also induced by the physician and not the person, so there should not be any co-payment” (male respondent, insured)*

## Discussion

Evidence related to health insurance is very scanty in Ethiopian context partly because health insurance was unavailable for large majority of the population [18]. However, health insurance is now key issue in the health sector reforms [25]. Therefore, our study contributes in providing information that can be used by policy makers. The use of qualitative research method ensured an in-depth investigation of the knowledge and preferences of the study participants regarding health insurance. Qualitative studies have been used to explore consumer preferences on different policy issues [29–31].

This study revealed that there are both demand and supply side factors which hindered the development of health insurance in the country. In the past, people were unaware of the health insurance benefit they were entitled to. Having little experience with health insurance, they are also found to be unfamiliar with some health insurance concepts though they have ideas about what health insurance is. There is little knowledge about the medical insurance that had been provided by commercial insurance corporations in the country. Most of the beneficiaries of these schemes are found to be employees of public enterprises, NGOs and other international organizations. The participants knew the new SHI proclamation though there is lack of detailed information.

Dissatisfaction with the current EBHI is observed for a variety of reasons such as limited benefit packages, retrospective reimbursement of healthcare expenses, cap, co-payments, and individual level enrolment. Some of the employees which are entitled to the services are not enrolled in some organizations for variety of reasons and discrimination between professionals and supportive staff in the same organization is said to be unacceptable.

The potential enrolees support SHI to be initiated to all formal sector employees in Ethiopia but they are concerned about the quality and availability of services. The key informants also raised quality as the main concern in the process of the initiation of the scheme. Though improvements in quality of services are cited in some of the government facilities they are still not satisfactory. Nevertheless, there is a need to initiate the scheme somewhere since out of pocket payments are unacceptable way of financing [32] and improvements can be achieved through time. No country has reached effective scheme at once [33, 34]. The experience of developed countries also shows that most of them are still far from achieving all of the objectives of SHI. But there is a need to clearly define the design of the scheme in order to ensure equity and sustainability. Though it is indicated that the people do not have the culture of going to health care [35] this may partly be due to payments; once insured that habit may change. Thus, the likely increase in service utilization following the introduction of SHI also needs to be considered in the design of the scheme. Otherwise, SHI may increase the problems of governing the health system rather than improving healthcare provision [36, 37].

Most of the participants of this study believe that it is the responsibility of the government to provide healthcare to its citizens. Thus, they should not have to contribute to the schemes. Their view is partly influenced by the current practice of providing health benefits free of charge by public enterprises and other organization. In the case of compulsory contributions, they opted for an average of 2–3 % of their gross monthly salary. Other studies also show that people opt for lower premium levels and co-payments [38]. Still, the premium offered increased with respect to quality of services and expansion of benefit packages. Participants of FGDs from public enterprises suggested higher contribution rates than the civil servants showing the income effect; that is, people with more income (salary) are willing to contribute a higher proportion of salary. Therefore, progressive contributions may be considered.

There is little understanding regarding benefit packages. Little knowledge of benefit packages is also observed in West Africa even among those who are covered by health insurance [8]. The participants for this study contend that every health problem should be treated in the same way as any illness prevents normal way of life. However, along the discussion, participants became aware of the scarcity of resources and the need for prioritization though they are not happy about it. Therefore, there will be a need for sensitization campaign in order to create general awareness regarding elements of health insurance.

There was also a view that there should be an incremental approach to benefit packages as adopted in other countries [33]. Consequently, the participants picked inpatient care, drugs, and emergency as very important benefit packages. Non-access to these services was indicated to lead in to catastrophic expenditure. This is comparable with a study in the USA among Maryland state employees which showed hospitalization coverage as the most important attribute for health insurance decision [39]. A study in India also revealed that respondent select drugs, inpatient, outpatient, and diagnostic tests as major benefits since they represent the highest expense [16, 39].

The participants were also aware that the disease burden of the country and the capacity of the health system be included in defining benefit packages. Some of the services are said to be available but they are not actually there. These are among the most important issues that need to be addressed while defining benefit packages [40, 41]. People can also forgo some of their personal needs to define benefits for everyone. In context of Ethiopia, emphasis is given to communicable disease at country level followed by non-communicable conditions. This shows that people can prioritize health care benefits depending on societal need or constraints. Effective prioritization of benefit packages under income constraints is also found elsewhere [42].

The experience of Germany shows a constant improvement in the benefit packages [33]. This is a lesson for other countries which are on process of initiating social health insurance. The country may start with limited benefit packages and use incremental approach as the pool increases, utilization changes, and economy grows. The expansion of benefit packages in other countries was also observed during economic growth [41].

The results of this study also revealed the frustration shared among the participants in terms of getting the right service. They may be forced to pay out of pocket even after being enrolled to SHI if services are not given by the provider of their choice. Other studies also revealed flexibility in the choice of doctors and choice of hospitals among the attributes in the decision making regarding health insurance [38, 39].

An interaction among the elements of the health insurance design was observed during the discussions. Respondents offered higher premium levels for increase in benefit packages and option of private provider or improvement in quality of services. The respondents have shown a preference for wide range of benefit packages. They opted for partial coverage of all services rather than full coverage of some expensive services. This is comparable with study from India which showed the preference for wide range of benefit packages at basic levels than a narrow benefit with higher coverage levels [16].

One of the issues in the current EBHI was retrospective reimbursements to individuals. If beneficiaries are still required to pay out of pocket at the point of service, it may still constitute burden of health care expenditure especially for those who do not readily have cash. Thus, there still is barrier to health care utilization. With regard to co-payments, the participants argue that it should not be applied on inpatient care since it is induced by physician and do not constitute moral hazard. Clear evidence in moral hazard is required to apply co-payments since it may reduce needed health care [40].

## Conclusions

Knowledge of concepts of health insurance such as risk poling and cost sharing is limited. In addition, there is a gap in awareness of health insurance proclamation, the elements, and progress of the policy. This may partly be due to reservations of study participants on the current condition of the health system and may not necessarily mean genuine lack of knowledge. Moreover, the participants of the study were not presented with the transcripts for feedback and comments and the chance of misinterpretation is not known. Though, the finding may not be generalized to the general public, it highlight areas that need to be considered in order to provide acceptable health insurance to the population and further research. In any case, there is need for further sensitization activities. Even though SHI is supposed to be compulsory, implementation would be easier if potential enrolees knew well how it works and what would be the benefits. Moreover, quality and availability of services are very important factors that should precede the initiation of the scheme.

## Abbreviations

EBHI: Employment based health insurance
FGDs: Focus group discussions
KIIs: Key informant interviews
NHA: National health accounts
MOH: Ministry of Health
SHI: Social health insurance
